# Comparative Analysis of the Use of Osteoplastic Materials in Socket Augmentation: A Systematic Review

**DOI:** 10.3390/biomimetics10110722

**Published:** 2025-10-29

**Authors:** Diana Sologova, Aida Kazaryan, Ilana Gor, Susanna Sologova, Elena Smolyarchuk, Ekaterina Grigorevskikh, George Anikin, Aida Mirzoeva, Khadi Albakova, Ekaterina Glazunova, Marina Skachkova, Pavel Petruk, Evgeny Presnyakov, Nasirzade Saba, Ekaterina Diachkova

**Affiliations:** 1 Department of Oral Surgery of Borovskiy Institute of Dentistry, Sechenov University, Mojaiskii val 11, 119048 Moscow, Russia; aya747@mail.ru (A.K.); gor_i_a@staff.sechenov.ru (I.G.); aida_mirzoevaDu@mail.ru (A.M.); khadialbakova@bk.ru (K.A.); katyusha.prokhorova@gmail.com (E.G.); marina.petukhova2014@ya.ru (M.S.); saba.nasirzade@gmail.com (N.S.); dyachkova_e_yu_1@staff.sechenov.ru (E.D.); 2 Department of Pharmacology, A. P. Nelyubin Institute of Pharmacy, I.M. Sechenov First Moscow State Medical University (Sechenov University), avenue Vernadsky 96/1, 119571 Moscow, Russia; sologova_s_s@staff.sechenov.ru (S.S.); smolyarchuk_e_a@staff.sechenov.ru (E.S.); grigorevskikh_e_m@staff.sechenov.ru (E.G.); 3 Department of Clinical Pharmacology and Propaedeutics of Internal Diseases, Sechenov University, B.Pirogovskaya 6/1, 119435 Moscow, Russia; anikin_g_s@staff.sechenov.ru; 4 Maxillofacial Surgery Department, I.M. Sechenov First Moscow State Medical University (Sechenov University), 8-2 Trubetskaya Str., 119991 Moscow, Russia; petruk_p_s@staff.sechenov.ru; 5 Russian Scientific Center of Surgery named after Academician B.V. Petrovsky, Scientific Research Institute of Human Morphology, Academician A.P. Avtsyna, 117418 Moscow, Russia; uvpres@gmail.com

**Keywords:** augmentation, guided bone regeneration, socket preservation, bone remodeling, biocompatibility, alveolar bone loss, bone resorption, tooth socket, bone regeneration, bone repair, bone substitute, biomaterials, systematic review

## Abstract

Background: Tooth extraction is often accompanied by bone tissue loss. This systematic review aims to compare osteoplastic materials for socket preservation. Methods: This systematic review was carried out with the PRISMA statement. To identify relevant studies, a thorough literature search was executed in several databases, such as Medline, PubMed, Scopus, and the Cochrane Central Register of Controlled Trials. The inclusion criteria were restricted to randomized controlled trials, and their methodological quality was evaluated with the Cochrane Risk of Bias tool (ROB 2). Results: Based on the predefined inclusion and exclusion criteria, nine randomized clinical trials published before 2024 were selected for analysis, all of them investigating the application of osteoplastic materials. Six studies performed xenogenic bone augmentation; one of them compared alloplastic and xenogenic materials; two studies described synthetic osteoplastic materials; and one described autogenic bone material. Conclusion: Socket augmentation with osteoplastic materials demonstrates effectiveness in preserving alveolar ridge dimensions and creating favorable conditions for further implant placement, though differences in clinical performance highlight the need for careful material selection.

## 1. Introduction

In order to obtain a predictable result in oral surgery after tooth extraction, various materials of natural and artificial origin are used. To avoid bone resorption, socket preservation and ridge preservation techniques are used after tooth extraction. Materials of animal origin (xenograft materials) are one of the commonly used materials in oral surgery. Nowadays, the use of natural systems, structures, and processes helps to solve scientific problems in medicine, including dentistry. One of the examples may be the application of xenograft materials in socket preservation and ridge preservation techniques [1,2,3].

Tooth extraction is performed if it is impossible to preserve the tooth by a conservative method of treatment. This is one of the most frequently performed procedures in oral surgery, which is accompanied by alveolar bone loss, an irreversible and progressive process due to physiological remodeling [4,5,6].

Bone loss involves both height and width, with loss in width up to 30% of the initial size in the period of first 3 months after the tooth extraction, 40% after 6 months, and up to 50% by 12 months after tooth extraction [7,8,9].

A decrease in the volume of the alveolar process after tooth extraction because of the natural process of bone remodeling primarily makes dental implant placement more challenging or even, in some cases, makes it impossible to restore the volume of the defect without any additional intervention, which endangers aesthetic and functional results [10,11].

The post-extraction healing process of the socket is clinically characterized by blood clot formation, followed by the development of a cortical bone bridge that seals the socket, ultimately resulting in epithelial coverage over the bone-filled site [12].

Considering the natural healing processes of the socket, various methods of regenerative preservation using bone grafts have been developed to prevent the resorption of alveolar bone after tooth extraction, improve aesthetics in the anterior region, and ensure optimal conditions for subsequent surgical interventions [13].

A range of biomaterials for bone transplantation, sourced from various origins, have been developed in response to the need for reconstructing resorbed bone. Traditional materials for bone defect regeneration are autogenous bone (from the person themselves), allogeneic (from another person) [14], xenogenic bone (in animals), and alloplastic, as well as the combination of resorbable and non-resorbable membranes to preserve the sockets [15,16]. Despite the fact that autogenous bone is the “gold standard” for regeneration of the bone, there are limitations, such as the additional field of surgery, the duration of surgery, a more difficult recovery period for the patient, the risk of complications, and the limited donor area, which motivate the search for alternative bone restoration materials [2,17].

In addition, various composite bone plastic materials are being developed that already contain collagen in their composition, which makes it possible to abandon the use of a collagen membrane to isolate the bone plastic material in the tooth socket [18].

Today, there are several basic techniques for alveolar ridge augmentation [9,19]:

− The technique of preserving the hole, which is carried out during tooth extraction. After tooth extraction, the intact socket well is filled with crushed bone plastic material, which can be an autograft, allograft, xenograft, or alloplastic material. The coronal part of the graft is usually covered with a membrane to help in its retention. In addition to preventing bone resorption, this technique also promotes earlier bone formation [20];
− Block transplantation is a method of increasing bone volume using a block that is fixed in the area of bone deficiency using screws. Autogenous bone grafts are the main material, but block allografts are also available, which are generally less predictable than autogenous grafts [20];
− The method of directed bone regeneration, which relies on the basics of guided tissue regeneration (GTR), according to which membrane is used to maintain space above the defect. In the case of GTR, this promotes the ingrowth of osteogenic cells and prevents the migration of unwanted cells from the overlying soft tissues. Resorbable and non-resorbable membranes are used for this method [21,22];
− A method of simultaneous implantation, in which a dental implant is installed in the tooth socket immediately after its removal [23,24].

Nonetheless, no material has been universally established as the standard for hole preservation, nor has a single technique been accepted as a standard for hole augmentation [25,26].

Several recent systematic reviews have examined techniques of socket preservation and the use of graft materials after tooth extraction [14]. However, most of them focus on a wide range of techniques and adjunctive tools (e.g., membranes, growth factors). In contrast, our review is specifically aimed at comparing the clinical effectiveness of different bone graft materials used for socket augmentation, providing an updated analysis based solely on recent clinical studies.

The aim of this systematic review is to critically evaluate and compare the clinical effectiveness of different osteoplastic materials used in socket augmentation, focusing on their impact on alveolar ridge preservation and subsequent implant development.

## 2. Materials and Methods

The current systematic review was designed and executed in alignment with the Preferred Reporting Items for Systematic Reviews and Meta-Analyses (PRISMA) framework, in addition to incorporating methodological standards set forth by Medline’s research instruments [27,28]. The completed PRISMA checklist is added to supplementary files.

The systematic review was prospectively registered with the International Prospective Register of Systematic Reviews (PROSPERO). The registration number is CRD42024605653.

### 2.1. Eligibility Criteria

The literature search in the current systematic review was structured according to the PICO framework, encompassing patient, intervention, comparison, and outcome elements.

Participant criteria: Patients from 18 to 80 years who have undergone tooth extraction.

Intervention types: Application of techniques aimed at alveolar ridge augmentation.

Control group: Comparisons included treatments performed without alveolar ridge augmentation techniques.

Outcome measures: Parameters assessing bone regeneration.

Study design: To ensure a high quality of evidence, the analysis was confined to peer-reviewed, English-language randomized controlled trials (RCTs). All other study designs and publication formats, such as conference abstracts and narrative reviews, were deemed ineligible.

### 2.2. Sources of Information

The literature search was performed across four electronic databases—Medline, PubMed, Scopus, and the Cochrane Central Register of Controlled Trials—covering publications up to the year 2024.

### 2.3. Search Strategy

The search terms describe the PECO components:

P (participants)—patients after tooth extraction;

E (exposure)—patients after tooth extraction and techniques for alveolar ridge augmentation;

C (comparison)—patients after tooth extraction who were treated without techniques for augmentation of the alveolar ridge;

O (outcome)—to assess the effectiveness of different osteoplastic materials for alveolar ridge augmentation in patients after extraction, the following criteria were used: radiographic measurement, histomorphological examination, and XRD analysis (X-Ray diffraction analysis).

Exclusion and Inclusion Criteria:

Inclusion criteria were as follows:

- Randomized clinical trials (RCTs);
- Participants aged between 18 and 80 years;
- Use of techniques for augmentation of the alveolar ridge.

Exclusion criteria were as follows:

- Other types of research;
- Participants under 18 years old and above 80 years old;
- Heavy smokers (>10 cigarettes per day);
- Severe comorbidity proof;
- Pregnancy and lactation;
- Other contraindications for tooth extraction.

Filters applied included full-text availability, clinical trials, RCTs, and publication dates from 2014 to 2024. Search algorithms were employed in Medline and PubMed. The search terms were used across all four databases (Medline, PubMed, Scopus, and Cochrane): «socket preservation», «tooth extraction», «osseointegration», «bone regeneration», «osteoplastic materials», «dental implantation», «oral cavity», «randomized clinical trials».

Medline/PubMed/Scopus/Cochrane («socket preservation» or «bone regeneration» or «osseointegration» or «osteoplastic materials» or «alveolar bone loss» or «bone resorption») AND («tooth extraction» or «dental implantation» or «tooth socket») AND («bone regeneration» or «bone repair»).

### 2.4. Selection of Studies

The selected included articles were from 2014 to 2024. Some researchers independently conducted database searches based on the specified criteria. Initially, 294 records were retrieved from various electronic databases. After excluding articles published before 2014 and removing duplicates, 98 studies remained. Subsequently, 20 manuscripts were excluded because of a lack of full-text availability and/or not being written in English, resulting in 87 studies. Further screening led to the exclusion of 79 articles due to insufficient relevant data, leaving a final total of 8 publications included in the review (Table 1).

### 2.5. Data Collection Process and Items

For each selected study, the following information was extracted: publication details (author, year, and journal), the design of the study, characteristics of participants (sample size, age, and comorbidities), and outcomes (changes in evaluated criteria used to assess the alveolar ridge effectiveness augmentation techniques in patients following tooth extraction). Particular emphasis was placed on the selection and clinical application of osteoplastic materials in the context of post-extraction socket augmentation.

Data extraction and collection were performed independently by three authors twice. Any discrepancies that arose were subsequently resolved through discussion.

### 2.6. Study Risk of Bias Assessment

A study was conducted with the Cochrane “Risk of Bias tool” (RoB 2.0) (Higgins 2019) [29] method:

− The randomization process;
− Deviations from intended interventions;
− Missing outcome data;
− Measurement of the outcome;
− Selection of the reported result.

The risk of bias was independently assessed by two authors using a standardized tool, with judgments categorized as low and high risk, or some concerns. Any disagreements regarding the bias assessment were resolved through discussion between the authors. Each item was assessed and documented accordingly in the risk of bias table.

**Table 1:** Characteristics of included studies “tooth extraction using osteoplastic materials”—randomized controlled trials.

Author, Year of Publication	(Total Number of Patients, in Each Group)	Age of Patients	Assessment Criterion	Protocols	Results
Monica Calasans-Maia [30] 2014	20	30–60	Histological sections Histomorphometrical evaluation using Image Pro-Plus	TG1: treated with the new bovine xenograft, Osseus, and TG2: treated with the established bovine xenograft, Bio-Oss	TG1, the volume of new bone-33.7 (±7.1), for CT − 32.3 (±8.9) for the remaining biomaterial-10.7 (±16.2). TG2, the volume of new bone-19.3 (±22.6), of the CT − 49.9 (±14.1) and of the remaining biomaterial-22.6 (±7.9).
Meloni et al. 2015 [31]	30 15 + 15	>18	RCT, studies on human, socket preservation, results	Socket sealing with connective tissue graft (group A) vs. porcine collagen matrix (group B)	Epithelial connective tissue graft: Vertical bone loss: 0.26 mm; Horizontal: 1.60 mm.Porcine collagen matrix: Vertical: 0.31 mm; Horizontal: 1.47 mm.
Marcelo Jose Uzeda, 2017 [32]	48 12 + 12 + 12 + 12	18–66	Clinical observations Histological analysis Histomorphometric evaluation XRD analysis	Clot (C), BoneCeramic (BC), Biomaterial 1 group (B1), and Biomaterial 2 group (B2).	B1 and B2 were less compact, with a roughness greater than that of BC. C group demonstrated newly formed bone interspersed with minor regions of connective tissue.
Renzo Guarnieri1 2017 [33]	30 10 + 10 + 10	>18	− The thickness of vestibular bone − Ridge width (RW) − The heights of the vestibular and lingual crest (HVC-HLC)	Group S: spontaneous healing. Group M: collagen membrane alone. Group GM: porcine-derived bone graft material associated with collagen membrane	The spontaneous healing group (S) exhibited the greatest resorption vertically (−2.13 mm) and horizontally (−3.96 mm). Sites treated with a collagen membrane alone (M) showed reduced resorption (−0.58 mm vertically; −0.91 mm horizontally). The most favorable outcomes were observed in sockets grafted with a porcine-derived bone and membrane (GM), with minimal vertical loss (−0.31 mm) and horizontal loss (−0.91 mm).
Maiorana et al. 2017 [8]	7 4 − F + 3 − M	>18	Histologic and histomorphometric analysis. Radiological evaluation	Demineralized bovine bone mineral covered with a porcine-derived noncrosslinked collagen matrix	1.21 mm—horizontally; 0.46 mm—vertically.
Márcio de Carvalho Formiga 2019 [4]	26 13 + 13	>18	Paired-t test to using Stata 14 CT scan	Group 1 (TG1; PTFE membrane + blood clot) Group 2 (TG2; PTFE membrane + xenogenic bone substitute biomaterial)	Buccal plate: control group—0.46 mm, test group—0.91 mm; Alveolar height: control group −0.41 mm, test group—0.35 mm
Manasi Yewale 2021 [21]	20 10 + 10	20–55	CBCT images	Group A—PRF Plus membrane and Sybograf plus ™ (70% HA and 30% β TCP) bone graft. Group B—Sybograf plus ™ (70% HA and 30% βTCP) bone graft	Quantitative assessments demonstrated that Group A experienced less vertical resorption (−1.48 mm) than Group B (−1.67 mm). While horizontal reduction in the width at 1, 3, and 5 mm depths was not statistically significant, Group A showed a superior gain in socket bone density at six months (1185.30 ± 473.21 HU) versus Group B (966.60 ± 273.27 HU).
Yuanyuan Sun, 2023 [17]	40 15 + 15 + 10	>18	Radiographic measurement Histomorphological examination Statistical analysis	Group 1: rhBMP-2/BioCaP/β-TCP Group 2: β-TCP Group 3: healing without material	Higher values of bone area in Group1.

## 3. Results

### 3.1. Study Selection

**Figure 1:**
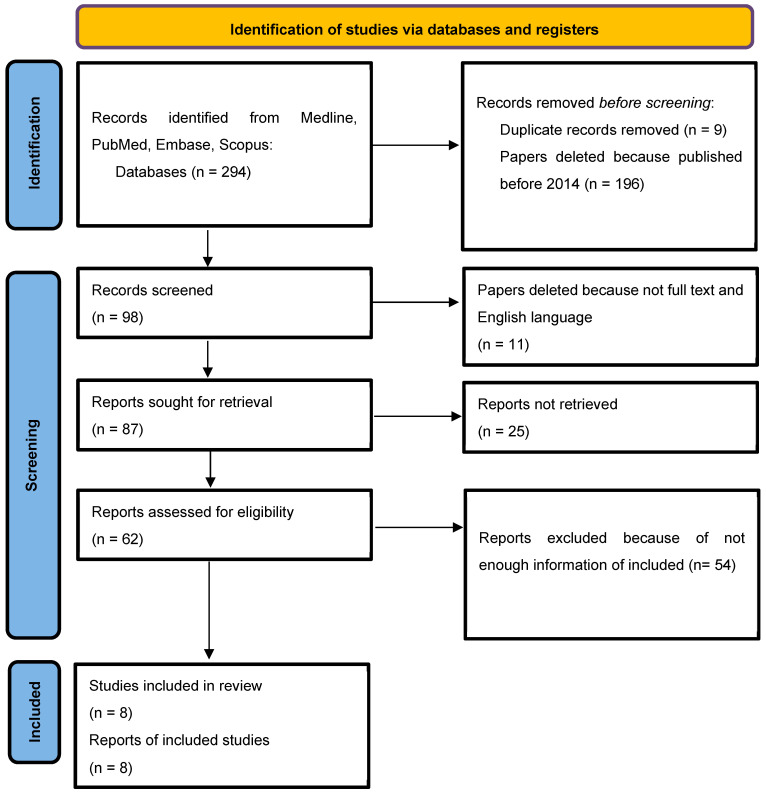
Flow diagram describing the selection process according to the Preferred Reporting Items for Systematic Reviews and Meta-Analyses (PRISMA) recommendations [27].

The research selection flow chart (Figure 1) [27] illustrates the initial identification of 294 articles retrieved from the PubMed database. A total of 9 articles were excluded due to repetitions, and another 196 articles were excluded because they were published before 2014. Then, 11 articles were deleted because they lacked the full text and were not published in English. Articles with insufficient information and non-compliant with our requirements were excluded from our systematic review results. Finally, eight articles were included in our study, which are devoted to the use of osteoplastic materials after tooth extraction.

### 3.2. Study Characteristics

Eight randomized controlled trials, published between 2014 and 2023, with a number of patients ranging from 7 to 48, with age groups varying from young adults (>18 years) to middle-aged individuals (up to 66 years) requiring tooth extraction and subsequent socket preservation, with periods varying from several months to one year, met the inclusion criteria for this review. All eight included studies involved participants free from comorbidities that might influence surgical outcomes, as well as those not taking medications that could impact postoperative healing.

Most trials evaluated xenogeneic osteoplastic materials, predominantly bovine-derived grafts such as Bio-Oss and Osseus [30], often applied in combination with collagen membranes. Several investigations assessed alloplastic substitutes, including β-tricalcium phosphate [17,21], biphasic calcium phosphate, and BoneCeramic, while one study used demineralized bovine bone combined with a porcine-derived collagen matrix. Autogenous grafts were not the primary focus of the included trials.

Outcome measures differed across studies and included histological and histomorphometric analyses, radiographic assessment with CBCT, and clinical evaluation of ridge dimensions. Despite variations in protocols, all studies reported that socket augmentation with osteoplastic materials reduced horizontal and vertical alveolar ridge resorption compared to ungrafted sites, xenografts provided the most predictable dimensional stability, and alloplasts showed favorable osteoconductive potential.

### 3.3. Risk of Bias Within Studies

All eight studies included in this systematic review were randomized controlled trials. The methodological quality of each was appraised using the Cochrane Risk of Bias 2 (RoB 2) tool, with the results detailed in Table 2.

## 4. Discussion

In recent years, the problem of maintaining the volume of the alveolar bone ridge after tooth extraction has remained one of the key issues in the dental world. This was confirmed by numerous randomized clinical trials published over the past ten years [30,34]. The amount of bone tissue affects the patient’s further treatment, including implant placement, aesthetic appearance, and restoration of chewing function [35,36]. The preservation of the alveolar bone also affects further prosthetic treatment [37].

Nowadays, there are some published articles about future directions for biomimetic or bioactive scaffolds application in socket and ridge preservation after extraction of the tooth in oral surgery [38,39]. A multiphase bioactive socket plug (BP) is designed to save the volume of sockets after tooth extraction in sockets with wall defects and to later provide sufficient bone volume for implantation [38]. In some studies, authors explore the osteogenic ability of bioactive glass (bioglass) with recombinant human bone morphogenetic protein-9 (rhBMP-9) in the bone regeneration process [39].

The healing process following tooth extraction results in considerable resorption of both the vertical and horizontal proportions of the alveolar ridge [40,41,42]. To prevent atrophy and reduce the percentage of loss of bone volume, there are various socket preservation techniques based on methods of intervention as well as the use of different osteoplastic materials [43]. To preserve the alveolar bone, an autograft, an allograft, and alloplastic and xenogenic bone material can be used.

In 2020, a study was conducted in which the augmentation of the tooth socket was performed using an allograft, and the results showed that the surgical field heals quite quickly; however, there is a high risk of complications, and late sequestration at the recipient site was also reported [44]. For this reason, the search for an alternative to this osteoplastic material continues.

Xenogenic bone material is produced in two types: in granules and blocks. The utilization of xenogeneic bone grafts presents a viable alternative to allogeneic products by mitigating their associated limitations and complications. Supporting this, a recent systematic review demonstrated that for the treatment of diverse bone defects, xenogeneic grafts yielded a similar increase in alveolar process width and comparable block survival rates to autogenous bone [45,46].

The effectiveness of autogenic material has been proven over many years of its use in clinical practice [47,48,49]. To date, this is the only transplant material for bone reconstruction containing native osteogenic cells. The advantages of this material include complete biocompatibility, lack of immunogenicity, and the ability to rapidly revascularize with the formation of organotypic bone tissue. The main disadvantages of autogenous transplants are that they require an additional operating area and there is a limited amount of donor material.

Alloplastic materials can be used due to their ease of handling, high formability, and sufficient absorption rate. Furthermore, their composition allows for combination of bioactive molecules, like growth factors, or with cell-based therapies to enhance regenerative outcomes [50,51,52].

A 2015 study conducted by several authors showed average histological results after a 3-month reintegration period, where the highest bone volume was preserved in sockets transplanted with alloplasts, followed by sockets without a graft, xenografts, and allografts [53].

Nowadays, the use of medical supplies that promote osteoinduction is becoming increasingly relevant and important, thereby arousing the interest of the entire medical community.

An ideal bone graft should have the following four characteristics: osteoconductivity (consists of the attraction and migration of osteoblasts, as well as the formation of a fibrin matrix), osteoinductivity (contains growth and regulation factors that stimulate bone formation), osteogenesis (contains cells that contribute to bone formation), and bone binding [54,55].

The results of clinical studies show excellent healing of soft tissues without loss of keratinized tissue and the absence of differences in changes in the marginal bone in the sockets after tooth extraction, as well as a high level of stability of primary implants. Preservation of the socket using minimally invasive surgical techniques ensures good healing of tissues, as well as the expected stability of implant placement in place of removed molars in severe periodontal diseases [56]. After tooth extraction, hard and soft tissues change in size. Many authors have conducted research to study biomaterials and surgical techniques used to preserve bone tissue. To guide optimal clinical practice, the biological effects and efficacy of socket preservation have been evaluated through multifaceted analyses, incorporating clinical, histological, volumetric, and molecular findings from the literature [57].

The evidence supports a protocol for predictable alveolar ridge preservation that integrates grafting materials, barrier membranes, and often tissue engineering or autogenous soft tissue grafts. Ultimately, the success of this regeneration is contingent upon a set of critical determinants: a population of osteoprogenitor cells, a stable and well-vascularized environment, space provision, and appropriate biochemical signals. Patient-specific factors remain a significant variable, contributing to the observed spectrum of osteoreparative results [58,59].

Grafts have a very important stimulating effect on osteogenesis on the part of the receiving bed. The research of the last five years indicates an increasing transition to the use of xenogenic and alloplastic materials [60].

The results of the current systematic review confirm that the use of osteoplastic materials of various origins (xenogenic, alloplastic, autogenic) can effectively slow down the processes of resorption and promote bone regeneration around the extracted tooth. It is important to consider that synthetic (alloplastic) materials in several studies have demonstrated better osteogenesis indicators compared to xenogenic analogues and are becoming more in-demand in modern clinical practice.

### Review Study Discussion

The aim of the current systematic review is to carefully analyze and integrate the available clinical evidence to evaluate the effectiveness of a variety of osteoplastic materials in maintaining the volume of the alveolar socket. It can be noted from the results obtained by cone-beam computed tomography scanning, histological examination, and clinical manifestations that almost every method had positive dynamics consisting of bone growth.

The eight randomized clinical trials presented in our table include many pieces of research related to the use of various osteoplastic materials and their further impact on bone regeneration. The studies were carried out during the removal of different groups of teeth followed by implantation. Materials of different origins were used, including xenogenic, alloplastic, and autogenic; comparison of these materials was carried out with cases without the use of additional means. Not only were the changes in the vertical and the horizontal bone loss evaluated but also the individual tolerance of each patient to the use of such drugs.

In several articles cited in our systematic review, the authors described the results obtained by many of them using xenogeneic materials, each of which showed significantly less bone loss compared to simple wound suturing [17]. However, in 2015, it was stated that, in addition to the use of bovine bone, it is reasonable to use pig collagen membranes [32]. Later, in 2017, augmentation with pig collagen and bone was also compared, and a decrease in bone in the group of patients without using the material was revealed [33]. This research is confirmed by the results of a subsequent article, in which a blood clot and a bovine graft were used in one group of patients. The second one proved to be more effective [4].

A major trial was conducted in 2022 which compared the use of xenogenic and allograft materials. Pig collagen showed the best result in measuring bone width. The results indicate that pig collagen showed the best result in measuring bone width. The effectiveness of other materials was proven by measuring vertical growth [8].

Referring to materials of other origins, we should also note their advantages. One is a natural autologous matrix enriched with platelets, which can be obtained using a simple blood sample and a natural centrifuge. It should be noted that postoperative pain levels did not differ between the Groups A and B, since patients experienced moderate pain. An assessment of postoperative swelling showed that only 30% of patients in Group A complained of it, while 80% of patients in Group B complained of postoperative edema. This material is widely used in surgical interventions and postoperative complications.

Autografts are universally recognized as possessing the most favorable biological properties for successful bone regeneration [21], and they have disadvantages in obtaining sufficient bone tissue from the donor site. Therefore, some authors have concluded that rhBMP-2/BioCaP/β-TCP, a synthetic material, is a promising bone substitute with a powerful proosteogenic effect, which can be used to preserve the socket in implantology, and it shows good results in bone formation.

Many modern scientists are inclined to use synthetic materials [29] based on calcium and its derivatives, but today, there are a small number of researchers devoted to this topic; xenogenic materials are used in almost every study, despite the ethical side of this issue.

However, despite the detailed consideration of this topic, we can note that our work has several limitations. Firstly, the review included articles that are not RCTs. This is due to the insufficient number of published RCTs on this topic. Secondly, despite generally positive clinical outcomes, heterogeneity in study design, evaluation methods, and follow-up periods prevents the identification of a single superior material.

## 5. Conclusions

This systematic review demonstrates that socket augmentation with osteoplastic materials is an effective approach to minimizing post-extraction alveolar ridge resorption and supporting optimal conditions for implant site development.

The included studies indicate that xenogeneic and alloplastic grafts provide predictable dimensional stability and favorable osteoconductive properties, whereas autogenous and allogeneic materials show good biological integration but may exhibit higher variability in resorption rates.

Overall, the evidence supports the application of osteoplastic materials as a reliable intervention for ridge preservation, while further high-quality RCTs with protocols and long follow-up periods are necessary to establish clear guidelines for optimal material selection. Teeth sockets using osteogenic materials are an effective method of preserving the volume of bone tissue after extraction of tooth, which significantly improves the prognosis of subsequent implantation and orthopedic treatment.

## Figures and Tables

**Table 2:** Risk of bias of randomized clinical trials, assessed through the ROB2 tool [29].

Study	The Randomization Process	Deviation from the Intended Interventions	Missing Outcome Data	Measurement of Outcome Data	Selection of the Reported Result
Monica Calasans-Maia, 2014 [30]	Low risk	Low risk	Low risk	Low risk	Low risk
Meloni et al., 2015 [31]	Low risk	Low risk	Low risk	Low risk	Low risk
Marcelo Jose Uzeda, 2017 [32]	Some concerns	Low risk	Low risk	Some concerns	Low risk
Renzo Guarnieri, 2017 [33]	Some concerns	Low risk	Low risk	Some concerns	Low risk
Maiorana et al., 2017 [8]	Some concerns	Low risk	Low risk	Some concerns	Low risk
Márcio de Carvalho Formiga, 2019 [4]	Low risk	Low risk	Low risk	Low risk	Low risk
Manasi Yewale, 2021 [21]	Low risk	Low risk	Low risk	Low risk	Low risk
Yuanyuan Sun, 2023 [17]	Low risk	Low risk	Low risk	Low risk	Low risk

## Data Availability

This study did not generate any new data; therefore, data sharing is not applicable.

## Supplementary Materials

The following supporting information can be downloaded at: https://www.mdpi.com/article/10.3390/biomimetics10110722/s1. PRISMA 2020 checklist [28].
