# Supplementary material for: Comparative Analysis of the Use of Osteoplastic Materials in Socket Augmentation: A Systematic Review
Source: Biomimetics (Basel). 2025 Oct 29;10(11):722. doi: 10.3390/biomimetics10110722 (PMC12650525; doi:10.3390/biomimetics10110722)
Supplement: Supplementary file 1 [file biomimetics-10-00722-s001.zip › biomimetics-3930555-supplementary.pdf]

PRISMA 2020 Checklist

This checklist has been completed for the manuscript entitled “Comparative Analysis Of The Use Of Osteoplastic Materials In The Socket Augmentation: A Systematic Review”. The checklist follows the PRISMA 2020 guidelines (http://www.prisma-statement.org).

Section and Topic	Item	Checklist item	Location /Comment
**TITLE**			
Title	1	Identify the report as a systematic review.	Title clearly identifies the study as a systematic review.
**ABSTRACT**			
Abstract	2	See the PRISMA 2020 for Abstracts checklist.	Abstract summarizes objectives, data sources (SCOPUS and WoS), and scope.
**INTRODUCTION**			
Rationale	3	Describe the rationale for the review in the context of existing knowledge.	Provided in the Introduction, pages 2-3
Objectives	4	Provide an explicit statement of the objective(s) or question(s) the review addresses.	Clearly stated in the end of introduction and in Materials and Methods -3rd page
**METHODS**			
Eligibility criteria	5	Specify the inclusion and exclusion criteria for the review and how studies were grouped for the syntheses.	Defined in Search Strategy-2.3
Information sources	6	Specify all databases, registers, websites, organisations, reference lists and other sources searched or consulted to identify studies. Specify the date when each source was last searched or consulted.	Explained in Search Strategy and Selection of Studies-Conducted in Medline, PubMed, Scopus, and Cochrane<br>Defined in Information sources-2.2
Search strategy	7	Present the full search strategies for all databases, registers and websites, including any filters and limits used.	The search terms describe the PECO components in the search strategy-2.3.<br>Pages 3-4
Selection process	8	Specify the methods used to decide whether a study met the inclusion criteria of the review, including how many reviewers screened each record and each report retrieved, whether they worked independently, and if applicable, details of automation tools used in the process.	Filtering described and illustrated in Fig. 1.<br>The selection process was defined in Selection of Studies-2.4.<br>4th page
Data collection process	9	Specify the methods used to collect data from reports, including how many reviewers collected data from each report, whether they worked independently, any processes for obtaining or confirming data from study investigators, and if applicable, details of automation tools used in the process.	Manual extraction and analysis<br>The Data collection process was clearly described in 2.5. Data Collection Process and Items<br>4th page
Data items	10a	List and define all outcomes for which data were sought. Specify whether all results that were compatible with each outcome domain in each study were sought (e.g. for all measures, time points, analyses), and if not, the methods used to decide which results to collect.	CVCT, histomorphometric analysis, keywords<br>4th page
	10b	List and define all other variables for which data were sought (e.g. participant and intervention characteristics, funding sources). Describe any assumptions made about any missing or unclear information.	The search terms describe the PECO components, they are described in 2.3. Search strategy<br>4th page
Study risk of bias	11	Specify the methods used to assess risk of bias in the included studies, including details of the tool(s) used, how many reviewers assessed each study and whether they worked independently,	The methodology of risk of bias assessment is described in

Section and Topic	Item	Checklist item	Location /Comment
assessment		and if applicable, details of automation tools used in the process.	2.6. Study Risk of Bias Assessment<br>5th page
Effect measures	12	Specify for each outcome the effect measure(s) (e.g. risk ratio, mean difference) used in the synthesis or presentation of results.	Not applicable
Synthesis methods	13a	Describe the processes used to decide which studies were eligible for each synthesis (e.g. tabulating the study intervention characteristics and comparing against the planned groups for each synthesis (item #5)).	Categorical and bibliometric analyses are described in the Methods.
	13b	Describe any methods required to prepare the data for presentation or synthesis, such as handling of missing summary statistics, or data conversions.	Analysis of risk of bias assessment in table 2.
	13c	Describe any methods used to tabulate or visually display results of individual studies and syntheses.	Analysis of risk of bias assessment in table 2.
	13d	Describe any methods used to synthesize results and provide a rationale for the choice(s). If meta-analysis was performed, describe the model(s), method(s) to identify the presence and extent of statistical heterogeneity, and software package(s) used.	Analysis of risk of bias assessment in table 2.
	13e	Describe any methods used to explore possible causes of heterogeneity among study results (e.g. subgroup analysis, meta-regression).	Analysis of risk of bias assessment in table 2.
	13f	Describe any sensitivity analyses conducted to assess robustness of the synthesized results.	Analysis of risk of bias assessment in table 2.
Reporting bias assessment	14	Describe any methods used to assess risk of bias due to missing results in a synthesis (arising from reporting biases).	The bias were assessed using “Risk of Bias tool” (RoB 2.0) (Higgins 2019) 2.6. Study Risk of Bias Assessment<br>5th page
Certainty assessment	15	Describe any methods used to assess certainty (or confidence) in the body of evidence for an outcome.	Not applicable
**RESULTS**			
Study selection	16a	Describe the results of the search and selection process, from the number of records identified in the search to the number of studies included in the review, ideally using a flow diagram.	Defined in 3.1. Study Selection (page 5)
	16b	Cite studies that might appear to meet the inclusion criteria, but which were excluded, and explain why they were excluded.	Defined in 3.1. Study Selection (page 5)
Study characteristics	17	Cite each included study and present its characteristics.	Defined in 3.2. Study Characteristics (page 8)
Risk of bias in studies	18	Present assessments of risk of bias for each included study.	The information about risk of bias is. Defined in Table 2 (page 8)
Results of individual studies	19	For all outcomes, present, for each study: (a) summary statistics for each group (where appropriate) and (b) an effect estimate and its precision (e.g. confidence/credible interval), ideally using structured tables or plots.	Results of individual studies are described in table 1 (pages 6-7)
Results of syntheses	20a	For each synthesis, briefly summarise the characteristics and risk of bias among contributing studies.	Results of syntheses are described in table 1 and 2 Pages (6,7,8)

Section and Topic	Item	Checklist item	Location /Comment
	20b	Present results of all statistical syntheses conducted. If meta-analysis was done, present for each the summary estimate and its precision (e.g. confidence/credible interval) and measures of statistical heterogeneity. If comparing groups, describe the direction of the effect.	Not applicable
	20c	Present results of all investigations of possible causes of heterogeneity among study results.	Not applicable
	20d	Present results of all sensitivity analyses conducted to assess the robustness of the synthesized results.	Not applicable
Reporting biases	21	Present assessments of risk of bias due to missing results (arising from reporting biases) for each synthesis assessed.	Biases are presented in table 2
Certainty of evidence	22	Present assessments of certainty (or confidence) in the body of evidence for each outcome assessed.	Not applicable
**DISCUSSION**			
Discussion	23a	Provide a general interpretation of the results in the context of other evidence.	The information is presented in 4.Discussion (Pages 9-10)
	23b	Discuss any limitations of the evidence included in the review.	The limitations are presented in the end of discussion (Page 11)
	23c	Discuss any limitations of the review processes used.	The limitations are presented in the end of discussion (Page 11)
	23d	Discuss implications of the results for practice, policy, and future research.	The limitations are presented in the end of discussion (Page 11)
**OTHER INFORMATION**			
Registration and protocol	24a	Provide registration information for the review, including register name and registration number, or state that the review was not registered.	The registration and protocol are presented in the beginning of 2. Materials and methods 3rd page
	24b	Indicate where the review protocol can be accessed, or state that a protocol was not prepared.	Not applicable
	24c	Describe and explain any amendments to information provided at registration or in the protocol.	Not applicable
Support	25	Describe sources of financial or non-financial support for the review, and the role of the funders or sponsors in the review.	Not applicable
Competing interests	26	Declare any competing interests of review authors.	The authors declare that there are no conflicts of interest. (Page 12)
Availability of data, code and other materials	27	Report which of the following are publicly available and where they can be found: template data collection forms; data extracted from included studies; data used for all analyses; analytic code; any other materials used in the review.	All data provided in the manuscript.
